# Systematic analysis and prediction for disease burden of ovarian cancer attributable to hyperglycemia: a comparative study between China and the world from 1990 to 2019

**DOI:** 10.3389/fmed.2023.1145487

**Published:** 2023-04-13

**Authors:** Peihong Wu, Qingtao Jiang, Lei Han, Xin Liu

**Affiliations:** ^1^Institute of Occupational Disease Prevention, Jiangsu Provincial Center for Disease Control and Prevention, Nanjing, China; ^2^Jiangsu Preventive Medicine Association, Nanjing, China; ^3^Department of Clinical Medicine, Jiangsu Health Vocational College, Nanjing, China

**Keywords:** ovarian cancer, hyperglycemia, disease burden, prediction, trend

## Abstract

**Background:**

Ovarian cancer is one of the most common female malignancies worldwide, and metabolic factors, such as hyperglycemia, are becoming potential risk factors. This study aimed to analyze the disease burden and its changing trend of ovarian cancer attributable to hyperglycemia in the Chinese population from 1990 to 2019.

**Methods:**

Using the data released by the Global Burden of Disease study 2019 (GBD 2019), we analyze the disease burden of ovarian cancer attributable to hyperglycemia in Chinese from 1990 to 2019 via morbidity, death, disability-adjusted life years (DALY); compare it with the global population; and predict the incidence and death trend in Chinese women for the next 10 years (2020–2029).

**Results:**

The incidence, death cases, and DALY numbers of ovarian cancer attributable to hyperglycemia in Chinese in 2019 were 2,751, 1,758, and 44,615 person-years, respectively, with an increase of 352.5%, 356.6%, and 329.0% compared with 1990, and the growth rate was higher than the global level. The age-standardized incidence rate (ASIR), age-standardized mortality rate (ASMR), and age-standardized DALY rate (ASDR) in 2019 were 0.270/100,000, 0.164/100,000, and 4.103/100,000, respectively. Moreover, the average annual percent changes (AAPCs) were 2.3%, 2.0%, and 2.0%, respectively, all higher than the global average. The disease burden of ovarian cancer attributable to hyperglycemia increased with age, reaching a peak in the 45–75 age group. The prediction of the neural network model showed that the incidence and death of the disease would remain high and rise in the next 10 years.

**Conclusion:**

The disease burden caused by ovarian cancer attributable to hyperglycemia in Chinese accounts for a large proportion globally, and the ASIR, ASMR, and ASDR are increasing year by year. We should continue to pay attention to the role of metabolic factors, such as hyperglycemia, in the occurrence and development of ovarian cancer, perform a good job in tertiary prevention, and strive to reduce health losses.

## Introduction

Ovarian cancer is one of the most common female malignancies worldwide. According to the latest global cancer statistics released in 2021, there are approximately 314,000 new cases of ovarian cancer every year (3.4% of all new cancer cases in women) and 207,000 ovarian cancer deaths (4.7% of all cancer deaths in women). The age-standardized incidence rate (ASIR) and the age-standardized mortality rate (ASMR) of ovarian cancer were 6.6/100,000 and 4.2/100,000, respectively (1). At present, there is no evaluative report on the comparative analysis and epidemiological prediction of the disease burden of ovarian cancer in China and globally.

In addition to genetic factors, such as BRCA mutations, non-genetic factors, such as diabetes mellitus (DM), high BMI, smoking, and alcohol drinking, are major risk factors for the development of ovarian cancer (2–4). Studies have confirmed that patients with risk factors, such as hyperglycemia, have a significantly increased vulnerability to ovarian cancer, and interventions with metformin or other antidiabetic agents can effectively reduce the incidence of ovarian cancer (5, 6). Systematic reviews have demonstrated that increased all-cause mortality and cancer-specific mortality in patients with ovarian cancer are associated with DM (7, 8). It is suggested that DM or hyperglycemia status is a potential hazard for ovarian cancer (9, 10). However, few studies have been conducted to inspect the disease burden of ovarian cancer attributable to metabolic factors and its trend, especially hyperglycemia-related ovarian cancer.

In this study, a series of data from the Global Burden of Disease study 2019 (GBD 2019) were used to reveal the relevant distribution information of ovarian cancer attributable to hyperglycemia in time, space, and population characteristics based on the disease burden indexes and to model and predict the disease trends in the next 10-year cycle, which is helpful to improve the allocation of health resources and policy formulation.

## Materials and methods

### Data source

The datasets derived from GBD 2019 provide the global burden of 369 diseases, injuries, and risk factors in 204 countries and regions worldwide (11). It can be obtained on the Global Health Data Exchange System website (GHDx, https://ghdx.healthdata.org). The ovarian cancer codes in GBD can correspond to ICD-9 (B123) and ICD-10 (C56) (12). The disease burden evaluation indexes used in this study include incidences, deaths, disability-adjusted life years (DALY), ASIR, ASMR, age-standardized DALY rate (ASDR), and average annual percent changes (AAPCs). The specific calculation methods can be referred to in the published research studies (13, 14). In addition, DALY in this study can be taken into account as both years of life lost (YLL) and years lived with disability (YLD) (15).

### Disease prediction

Back propagation neural network is a multilayer feedforward network trained by an error back propagation algorithm (16). Using the current state and historical data of the research object as input, the error between the output predicted value and the actual value decreases along the gradient direction by repeatedly training and adjusting the connection weights and thresholds in the neural network, and the network parameters with the smallest error are determined to achieve the purpose of predicting the future state (17). The data on ovarian cancer attributable to hyperglycemia from 1990 to 2019 were applied to conduct the model. According to the training pattern of the BP neural network, the input layer has 10 nodes, the hidden layer has 10 nodes, and the hidden layer activation function is tansig. The number of output layer nodes is 1, and the activation function of the output layer is logsig. The learning rate is set to 0.05, and the convergence error is 0.005.

### Statistical analysis

A series of estimates, such as the number of incidences, were expressed by their 95% uncertainty intervals (UIs) calculated by generating 1,000 samples at each computational step and taking the ordinal values of the 25th and 975th values (18). The trend analysis of the rate was expressed by AAPC, which was generated and analyzed by the Joinpoint software (V 4.7.0.0), with α = 0.05 as the test level (19). The R software (V 4.0.3) dplyr and ggplot2 packages were employed for data cleaning and visualization, and the AMORE package was used for neural network forecasting.

## Results

### Incidence and death of ovarian cancer attributable to hyperglycemia in China and worldwide

**Figure 1:**
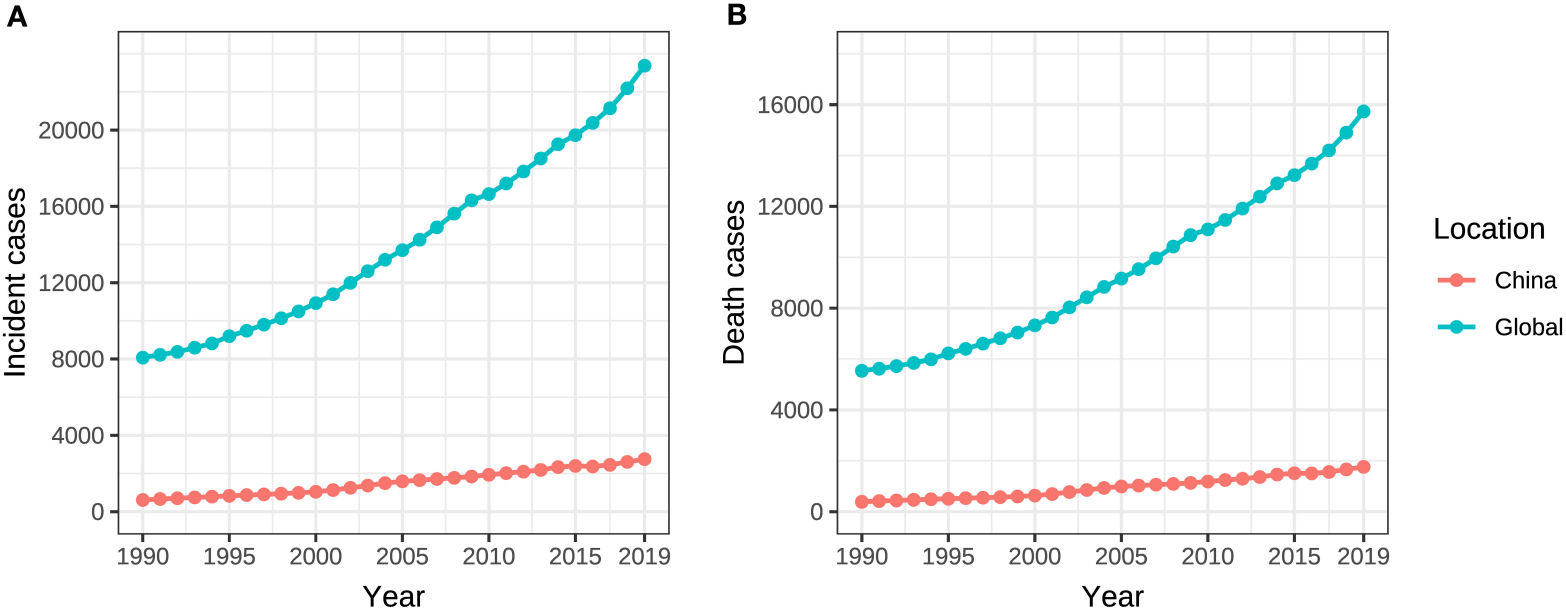
Incident and death cases of ovarian cancer attributable to hyperglycemia from 1990 to 2019. **(A)** Incident cases, **(B)** Death cases.

From 1990 to 2019, the incident and death cases of ovarian cancer in Chinese women were summed up as 45,920 and 28,564, respectively, accounting for 10.9% and 10.1% of the incidences (422,385) and deaths (283,500) among the global population during the same period. The incident and death cases of ovarian cancer attributable to hyperglycemia in Chinese women increased from 608 (95% UI: 475–840) and 385 (95% UI: 70–1,024) in 1990 to 2,751 (95% UI: 2,003–3,470) and 1,758 (95% UI: 299–4,429) in 2019. Globally, the incidence and death rose from 8,070 cases (95% UI: 7,434–9,156) and 5,537 cases (95% UI: 1,096–13,331) to 23,376 cases (95% UI: 20,695–26,179) and 15,736 cases (95% UI: 3,023–36,227), with an increase of 189.7% and 184.2%, respectively (Table 1, Figure 1).

**Table 1:** Incidence and death of ovarian cancer attributable to hyperglycemia from 1990 to 2019.

**Location**	**Year**	**Incident cases**	**ASIR (×1/10^5^)**	**Death cases**	**ASMR (×1/10^5^)**
China	1990	608	0.132	385	0.090
	1995	822	0.157	505	0.105
	2000	1,037	0.174	627	0.112
	2005	1,585	0.233	987	0.152
	2010	1,930	0.245	1,181	0.154
	2015	2,394	0.263	1,508	0.164
	2019	2,751	0.270	1,758	0.164
	**Change rate (%)**	352.5	104.5	356.6	82.2
	**AAPC (%)**		2.3 (2.0–2.7)		2.0 (1.6–2.5)
	***t***		13.7		8.5
	***P***		< 0.001		< 0.001
Global	1990	8,070	0.376	5,537	0.266
	1995	9,195	0.384	6,220	0.268
	2000	10,928	0.408	7,322	0.282
	2005	13,710	0.455	9,163	0.311
	2010	16,645	0.486	11,095	0.329
	2015	19,735	0.506	13,229	0.339
	2019	23,376	0.541	15,736	0.359
	**Change rate (%)**	189.7	43.9	184.2	35.0
	**AAPC (%)**		1.3 (1.1–1.5)		1.1 (0.9–1.2)
	***t***		16.2		13.1
	***P***		< 0.001		< 0.001

**Figure 2:**
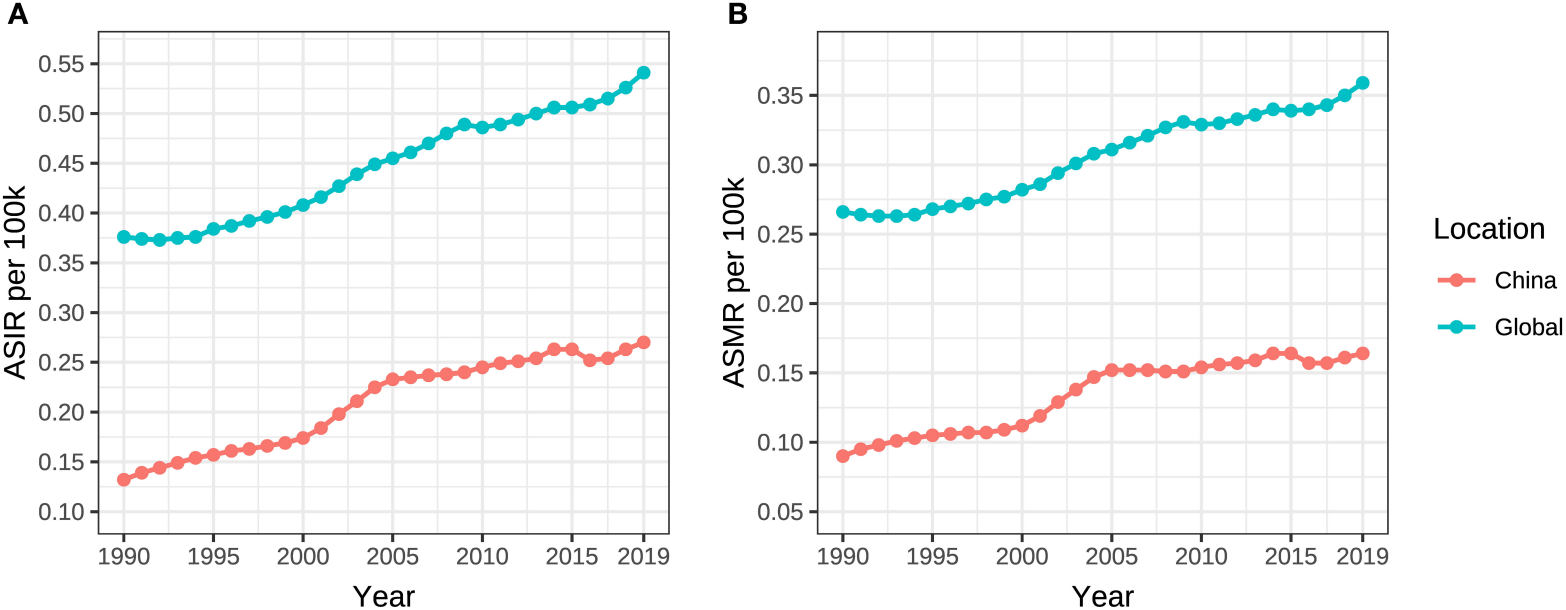
ASIR and ASMR of ovarian cancer attributable to hyperglycemia from 1990 to 2019. **(A)** ASIR, **(B)** ASMR.

**Figure 3:**
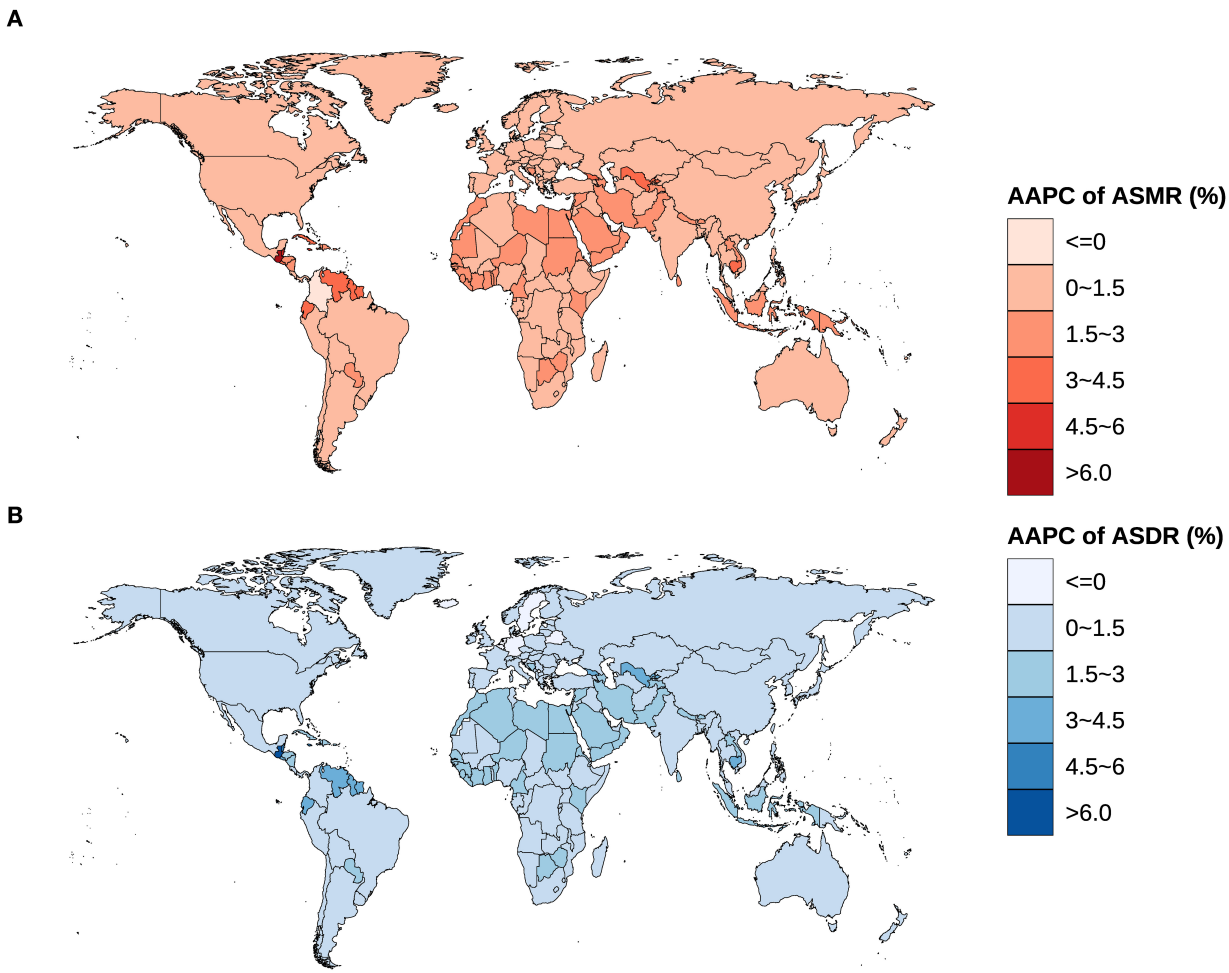
Global spatial distribution of AAPCs on ASMR and ASDR of ovarian cancer attributable to hyperglycemia from 1990 to 2019. **(A)** AAPC of ASMR, **(B)** AAPC of ASDR.

The ASIR and ASMR of ovarian cancer attributable to hyperglycemia in Chinese women grew from 0.132/100,000 (95% UI: 0.103/100,000–0.184/100,000) and 0.090/100,000 (95% UI: 0.016/100,000–0.239/100,000) in 1990 to 0.270/100,000 (95% UI: 0.198/100,000–0.339/100,000) and 0.164/100,000 (95% UI: 0.028/100,000–0.414/100,000) in 2019 with AAPCs as 2.3% and 2.0% (*P* < 0.001), respectively. Globally, the ASIR and ASMR increased from 0.376/100,000 (95% UI: 0.347/100,000–0.424/100,000) and 0.266/100,000 (95% UI: 0.053/100,000–0.642/100,000) to 0.541/100,000 (95% UI: 0.478/100,000–0.606/100,000) and 0.359/100,000 (95% UI: 0.069/100,000–0.826/100,000) with AAPCs as 1.3% and 1.1% (*P* < 0.001), respectively (Table 1, Figure 2). In addition, the AAPC of ASMR in countries around the world is presented in Figure 3A.

### Disease burden caused by ovarian cancer attributable to hyperglycemia

In 2019, DALY caused by ovarian cancer attributable to hyperglycemia in Chinese women was 44,615 person-years, accounting for 0.026% of the disease burden of all causes in Chinese women (168,758,861 person-years) and 12.6% of the DALY induced by ovarian cancer attributable to hyperglycemia in the global population (353,748 person-years).

**Figure 4:**
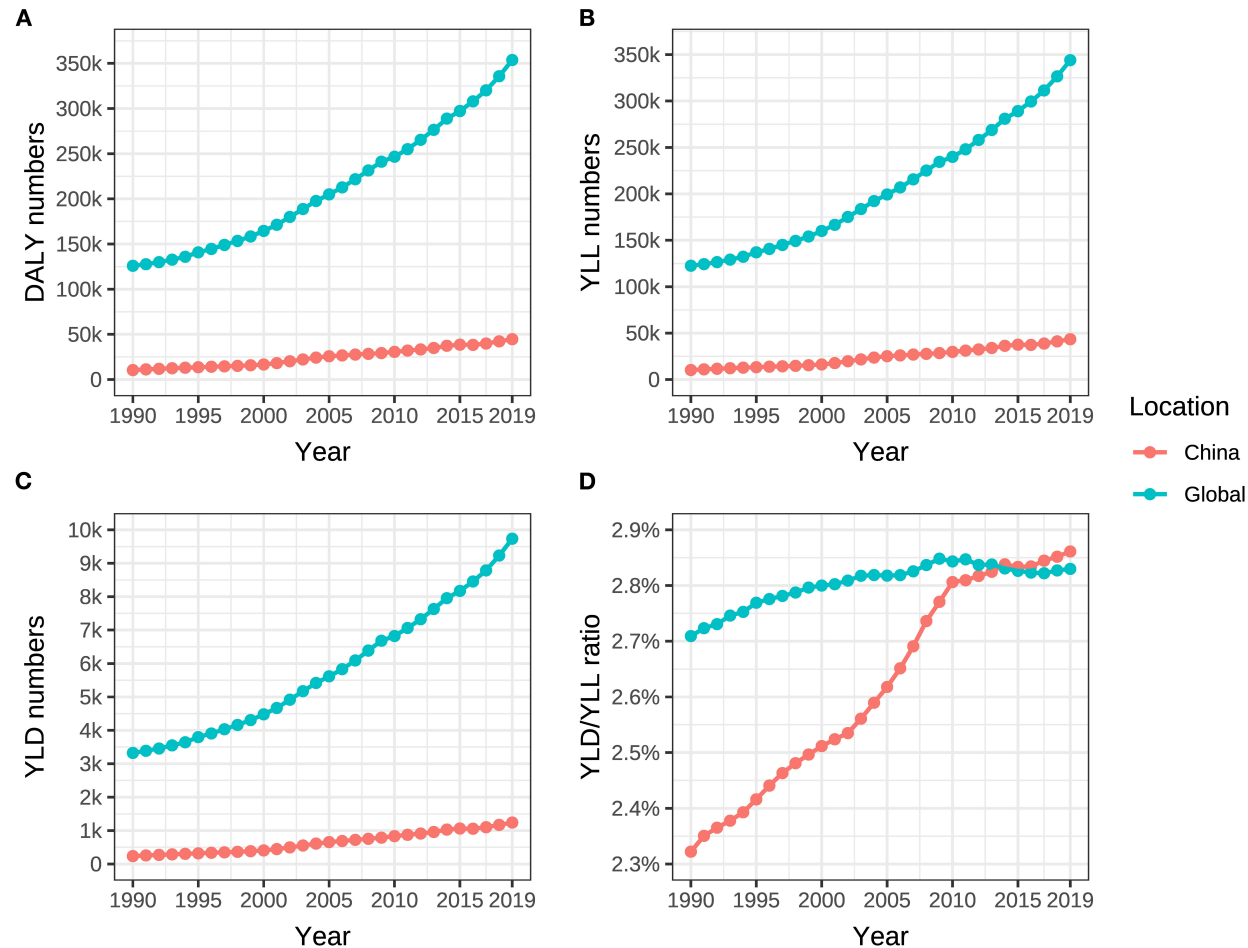
DALY, YLL, YLD of ovarian cancer, and YLD/YLL ratio attributable to hyperglycemia from 1990 to 2019. **(A)** DALY numbers, **(B)** YLL numbers, **(C)** YLD numbers, and **(D)** YLD/YLL ratio.

In China, DALY, YLL, and YLD due to ovarian cancer attributable to hyperglycemia increased from 10,399 (95% UI: 1,883–27,641), 10,163 (95% UI: 1,844–26,898), and 236 person-years (95% UI: 40–636) in 1990 to 44,615 (95% UI: 7,595–113,363), 43,374 (95% UI: 7,438–110,204), and 1,241 person-years (95% UI: 194–3,294) in 2019, respectively. During the same period, DALY, YLL, and YLD in the whole world changed from 125,945 (95% UI: 24,615–304,316), 122,623 (95% UI: 23,982–296,166), and 3,322 person-years (95% UI: 587–8,277) to 353,748 (95% UI: 68,513–824,409), 344,013 (95% UI: 65,804–800,804), and 9,735 person-years (95% UI: 1,774–23,919), respectively. The growth rates of DALY, YLL, and YLD in China (329.0%, 326.8%, and 425.8%) were much higher than those in the world (180.9%, 180.5%, and 193.0%) (Table 2, Figures 4A–C). YLL was always the dominance of DALY, but the YLD/YLL ratio of ovarian cancer attributable to hyperglycemia in Chinese women showed an increasing trend from 1990 to 2019, with an ascent (0.539%) higher than the global level of 0.121%, as shown in Table 2, Figure 4D.

**Table 2:** Disease burden of ovarian cancer attributable to hyperglycemia from 1990 to 2019.

**Location**	**Year**	**DALY (person-years)**	**ASDR (×1/10^5^)**	**YLL (person-years)**	**ASYLLR (×1/10^5^)**	**YLD (person-years)**	**ASYL (×1/10^5^)**	**YLD/YLL (%)**
China	1990	10,399	2.292	10,163	2.240	236	0.052	2.322
	1995	13,522	2.632	13,203	2.570	319	0.062	2.416
	2000	16,572	2.793	16,165	2.725	406	0.068	2.512
	2005	25,714	3.749	25,058	3.653	656	0.096	2.618
	2010	30,554	3.793	29,720	3.690	834	0.103	2.806
	2015	38,543	4.045	37,481	3.934	1,062	0.112	2.833
	2019	44,615	4.103	43,374	3.988	1,241	0.115	2.861
	**Change rate (%)**	329.0	79.0	326.8	78.0	425.8	121.2	0.539
	**AAPC (%)**		2.0 (1.5–2.4)		2.0 (1.5–2.4)		2.6 (2.3–2.9)	
	***t***		8.9		8.8		15.3	
	***P***		< 0.001		< 0.001		< 0.001	
Global	1990	125,945	5.890	122,623	5.735	3,322	0.156	2.709
	1995	140,885	5.946	137,089	5.786	3,796	0.160	2.769
	2000	164,450	6.211	159,971	6.041	4,479	0.169	2.800
	2005	204,960	6.868	199,343	6.680	5,617	0.188	2.818
	2010	246,685	7.240	239,865	7.040	6,820	0.200	2.843
	2015	297,271	7.585	289,100	7.377	8,171	0.209	2.826
	2019	353,748	8.091	344,013	7.868	9,735	0.223	2.830
	**Change rate (%)**	180.9	37.4	180.5	37.2	193.0	42.9	0.121
	**AAPC (%)**		1.1 (1.0–1.3)		1.1 (1.0–1.3)		1.3 (1.1–1.5)	
	***t***		15.6		15.5		15.6	
	***P***		< 0.001		< 0.001		< 0.001	

The standardized DALY, YLL, and YLD rates (ASDR, ASYLLR, and ASYLDR) of ovarian cancer attributable to hyperglycemia in China elevated from 2.292/100,000 (95% UI: 0.416/100,000–6.053/100,000), 2.240/100,000 (95% UI: 0.407/100,000–5.931/100,000), and 0.052/100,000 (95% UI: 0.009/100,000–0.142/100,000) in 1990 to 4.103/100,000 (95% UI: 0.701/100,000–10.418/100,000), 3.998/100,000 (95% UI: 0.686/100,000–10.126/100,000), and 0.115/100,000 (95% UI: 0.018/100,000–0.306/100,000) in 2019, respectively, while that in the global population ascended from 5.890/100,000 (95% UI: 1.152/100,000–14.213/100,000), 5.735/100,000 (95% UI: 1.122/100,000–13.843/100,000), and 0.156/100,000 (95% UI: 0.028/100,000–0.388/100,000) in 1990 to 8.091/100,000 (95% UI: 1.566/100,000–18.863/100,000), 7.868/100,000 (95% UI: 1.504/100,000–18.316/100,000), and 0.223/100,000 (95% UI: 0.041/100,000–0.548/100,000) in 2019, respectively.

**Figure 5:**
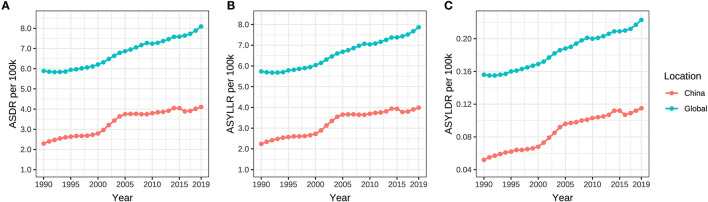
**(A–C)** ASDR, ASYLLR, and ASYLDR of ovarian cancer attributable to hyperglycemia from 1990 to 2019.

The ASDR, ASYLLR, and ASYLDR of ovarian cancer attributable to hyperglycemia increased by 79.0%, 78.0%, and 121.2%, respectively, in China, which was substantially higher than those (37.4%, 37.2%, and 42.9%) of the world. Meanwhile, the AAPCs of ASDR, ASYLLR, and ASYLDR in Chinese women (2.0%, 2.0%, and 2.6%) were also higher than those in the global population (1.1%, 1.1%, and 1.3%) (Table 2, Figure 5). The map delineating the AAPC of ASDR in countries worldwide is shown in Figure 3B.

### Age distribution of disease burden caused by ovarian cancer attributable to hyperglycemia

**Figure 6:**
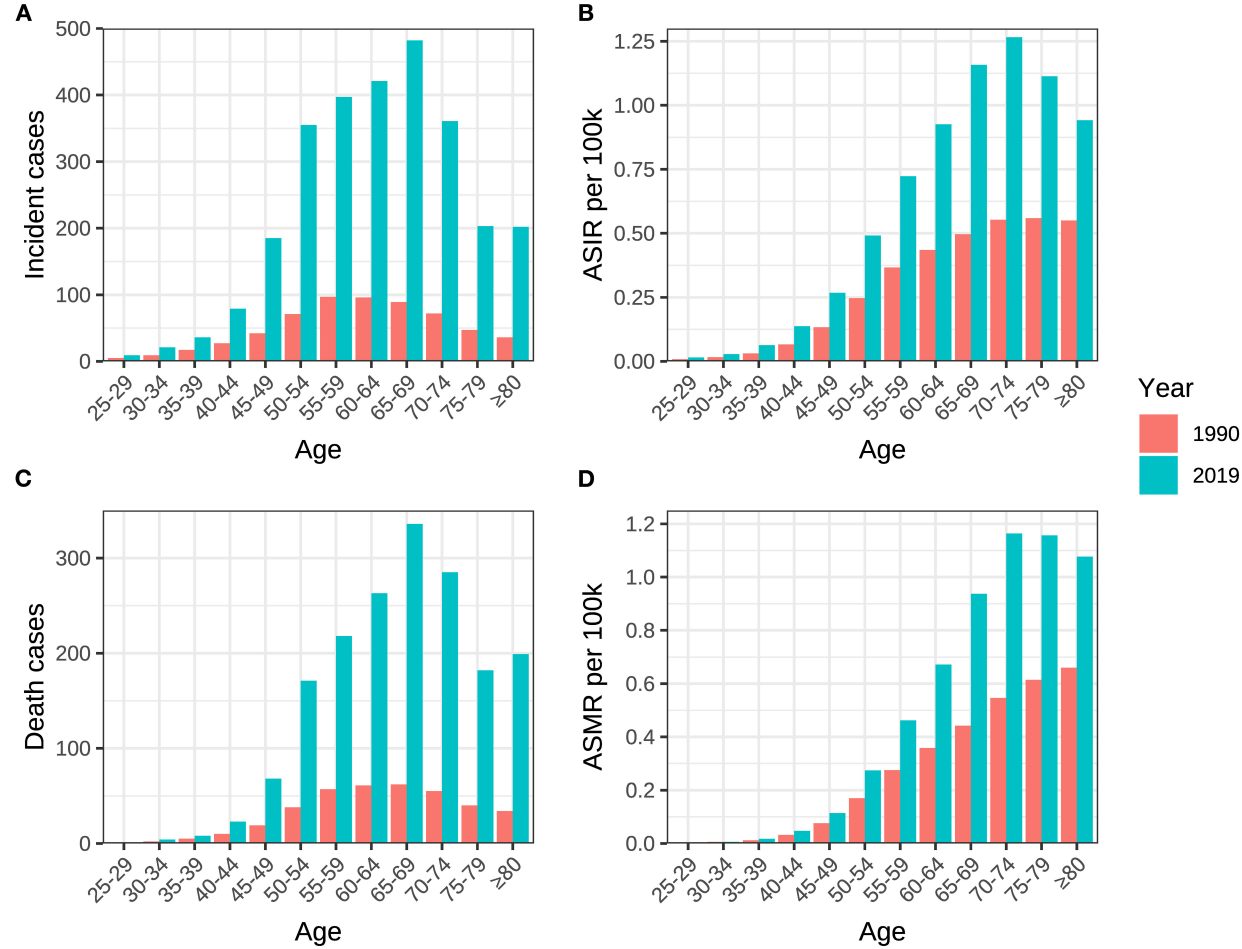
Age distribution of incidence, death, ASIR and ASMR of ovarian cancer attributable to hyperglycemia in China between 1990 and 2019. **(A)** Incident cases, **(B)** ASIR, **(C)** death cases, and **(D)** ASMR.

The incidence, mortality, and DALY of ovarian cancer attributable to hyperglycemia were all at a relatively low level between 25 and 44 years old and began to rise rapidly after 45, reaching a peak between age groups of 55 and 74. It could be distinguished from the year distribution that morbidity and mortality in 1990 did not show a downward trend after the age of 75. However, in 2019, all disease burden indicators revealed a significant decline in age groups beyond 75 (Tables 3, 4, Figure 6).

**Table 3:** The age distribution on ASRs of ovarian cancer attributable to hyperglycemia in China, 1990.

**Age**	**Incident cases**	**ASIR (×1/10^5^)**	**Death cases**	**ASMR (×1/10^5^)**	**DALY (person-years)**	**ASDR (×1/10^5^)**
25–29	5	0.008	1	0.002	67	0.125
30–34	9	0.016	2	0.006	136	0.322
35–39	17	0.031	5	0.012	283	0.641
40–44	27	0.066	10	0.032	497	1.554
45–49	42	0.133	19	0.076	797	3.264
50–54	71	0.247	38	0.170	1,450	6.446
55–59	97	0.367	58	0.275	1,885	9.117
60–64	96	0.435	61	0.358	1,750	10.187
65–69	89	0.496	62	0.442	1,470	10.566
70–74	72	0.553	55	0.546	1,079	10.673
75–79	47	0.559	40	0.614	618	9.503
≥80	36	0.550	34	0.660	366	7.127
Total	608	0.132	385	0.090	10,399	2.292

**Table 4:** The age distribution on ASRs of ovarian cancer attributable to hyperglycemia in China, 2019.

**Age**	**Incident cases**	**ASIR (×1/10^5^)**	**Death cases**	**ASMR (×1/10^5^)**	**DALY (person-years)**	**ASDR (×1/10^5^)**
25~29	9	0.015	1	0.002	87	0.159
30~34	21	0.028	4	0.006	244	0.383
35~39	36	0.063	8	0.017	443	0.894
40~44	79	0.137	23	0.047	1,133	2.278
45~49	185	0.268	68	0.114	2,945	4.947
50~54	355	0.491	171	0.274	6,518	10.469
55~59	397	0.723	218	0.462	7,255	15.372
60~64	421	0.926	263	0.672	7,502	19.190
65~69	482	1.158	336	0.937	8,026	22.396
70~74	361	1.266	285	1.164	5,575	22.727
75~79	203	1.113	182	1.157	2,800	17.825
≥80	202	0.942	199	1.077	2,087	11.290
**Total**	2 751	0.270	1,758	0.164	44,615	4.103

### Prediction of incidence and mortality of ovarian cancer attributable to hyperglycemia

**Figure 7:**
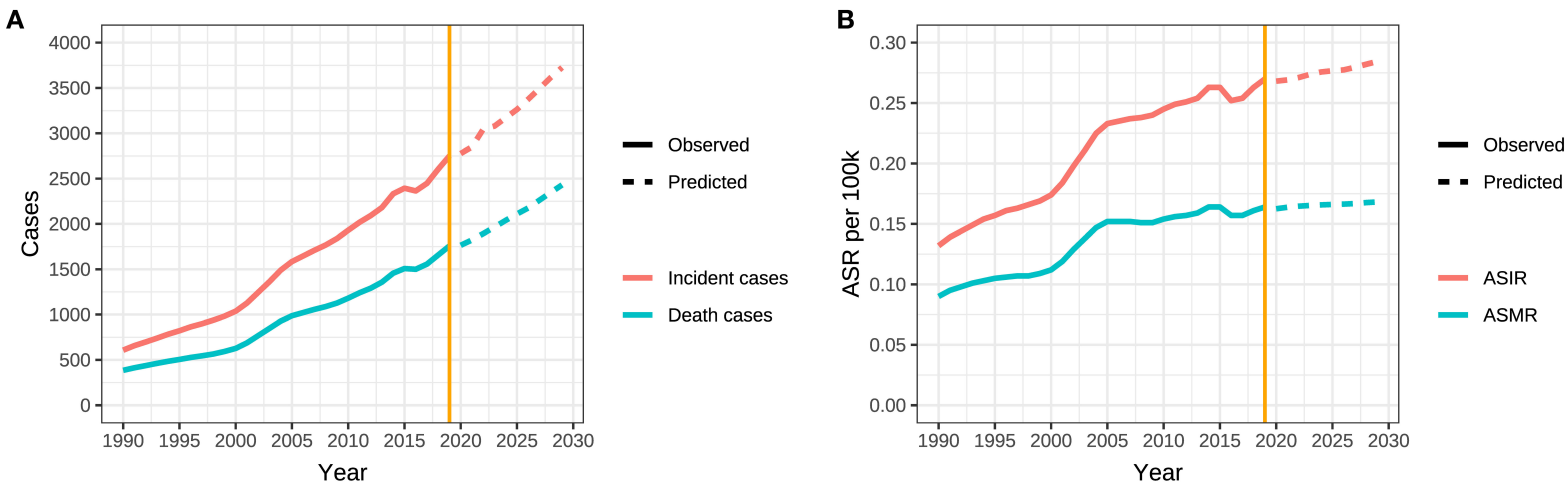
Trends in observed and predicted incidence, death, ASR of ovarian cancer attributable to hyperglycemia from 1990 to 2029 in China. **(A)** Trends in observed and predicted incidence and death and **(B)** Trends in observed and predicted ASR.

The BP neural network model with fine goodness of fit was selected to predict the incidence and mortality trend of ovarian cancer attributable to hyperglycemia in China from 2020 to 2029. It was forecasted that the incidence and mortality status of ovarian cancer attributable to hyperglycemia in Chinese women would continue to rise in the next 10-year cycle. The number of morbidity and death was likely to reach 3,726 and 2,430 in 2,029, respectively, and ASIR and ASMR might reach 0.284/100,000 and 0.168/100,000 in 2029 (Table 5, Figure 7).

**Table 5:** Incidence and death of ovarian cancer attributable to hyperglycemia in China predicted by neuro-network model from 2020 to 2029.

**Year**	**Incident cases**	**ASIR (×1/10^5^)**	**Death cases**	**ASMR (×1/10^5^)**
2020	2,777	0.268	1,765	0.163
2021	2,850	0.269	1,824	0.164
2022	3,045	0.271	1,893	0.165
2023	3,079	0.274	1,965	0.165
2024	3,171	0.276	2,039	0.166
2025	3,263	0.277	2,110	0.166
2026	3,376	0.277	2,176	0.166
2027	3,494	0.280	2,262	0.167
2028	3,616	0.282	2,347	0.168
2029	3,726	0.284	2,430	0.168

## Discussion

Globally, the incidence, mortality, and DALY of ovarian cancer are on the rise. The disease burden in South Asia, East Asia, and Western Europe is the heaviest, among which China and India have the most death cases (20, 21). In addition to genetic and reproductive factors, the onset of ovarian cancer is also closely related to metabolic risks, such as obesity, high BMI, and high blood glucose levels, caused by insulin resistance, all of which are supported profoundly by relevant basic research (22–24).

More attention has been paid to the pathogenesis of ovarian cancer associated with high fasting plasma glucose. From the perspective of energy supply, high glucose promotes tumor cell proliferation (25). In addition, cancer cell mitosis could be promoted by hyperinsulinemia caused by insulin resistance through molecules such as insulin receptor-A and insulin-like growth factor-1 (IGF-1), and high glucose could activate the IGF-1 receptor signaling pathway (26–28). Meanwhile, changes in blood glucose levels have significant impacts on signal transduction in cancer cells (29, 30). It has been illustrated that diabetes mellitus can also promote carcinogenesis through the regulation of programmed cell death and immune system surveillance (31, 32). Currently, there has been no in-depth report on the disease burden of ovarian cancer attributable to hyperglycemia.

Though the disease burden of ovarian cancer attributable to hyperglycemia is not serious compared to specific risk factors, such as genetics and behavior in Chinese and world populations, the rising trend is not optimistic. ASIR and ASMR in China increased from 0.132/100,000 and 0.090/100,000 in 1990 to 0.270/100,000 and 0.164/100,000 in 2019, with AAPCs of 2.3% and 2.0%, respectively, much higher than the global rates of 1.3% and 1.1%. The average annual growth rates of ASDR, ASYLLR, and ASYLDR among Chinese women (2.0%, 2.0%, and 2.6%) were all beyond the global levels (1.1%, 1.1%, and 1.3%). The ratio of YLD/YLL in China rose from 2.322% in 1990 to 2.861% in 2019, with an increase of 0.539%, which also exceeded 0.121% globally during the same period. The results indicate that great progress in the diagnosis and treatment of ovarian cancer has been made in China, and the early detection and upgrade of regimens have extended the survival of patients and improved the prognosis. Nonetheless, the vast majority of DALY for hyperglycemia-related ovarian cancer still comes from YLL.

Our results unveiled that the incidence and mortality of ovarian cancer attributable to hyperglycemia ascended with age, and ASIR increased most rapidly in the 45–75 group, which was consistent with the conclusions of other research studies, suggesting that more attention should be paid to the perimenopausal and elderly people (33, 34). Based on the understanding of the incidence trend of ovarian cancer attributable to hyperglycemia in the past 30 years, our prediction was made in combination with the current observed trend in this study. The charts indicate that although the epidemic trend of ovarian cancer is decreasing gradually, the overall rising potential cannot be ignored. In addition, with the extension of aging, the absolute number of morbidity and mortality will be always at a high level in the next 10-year cycle.

Considering the dismal etiology and high malignancy of hyperglycemia-related ovarian cancer, it is still necessary to attach great importance to reducing the occurrence of abnormal glucose metabolism, such as diabetes, and improving the blood glucose status of middle-aged and elderly women. First, it is necessary to arouse people's concerns about the high risk of hyperglycemia through scientific popularization, which can help people develop good living habits by reducing sugar consumption and increasing activity. Meanwhile, the key point to control ovarian cancer is to strengthen the propaganda of clinical symptoms associated with ovarian cancer for the general population, such as abdominal distension, abdominal pain, and increased abdominal circumference. In other words, people need to pay more attention to body changes in daily life. In terms of high-risk population, especially people with a family history of ovarian cancer, early screening of ovarian cancer can be taken into consideration including vaginal ultrasonography and the monitoring of serum carbohydrate antigen 125 (CA125), so as to diagnose and treat in time and ameliorate the survival of patients.

## Conclusion

Compared with traditional genetic factors, this study focuses on the non-genetic factor, hyperglycemia for the development of ovarian cancer. More attention is paid to the disease burden and its development trend caused by ovarian cancer attributable to hyperglycemia. It is revealed that both incidence and mortality are on the rise and the upward trend will continue over the next decade in China and worldwide. The indicators related to the disease burden maintain a high level between 55 and 74 years old. It is suggested to conduct further screening on the susceptibility of ovarian cancer for people with hyperglycemia. The limitation of this study is that some of the data in the GBD study are based on statistical calculations. Affected by algorithms and models, the integrity of the data may cause certain deviations. Moreover, DALY is influenced by social culture and disease cognition, which has certain drawbacks and may not fully reflect the native situation (35, 36). Due to the lack of relevant data, this study also failed to analyze the economic burden caused by ovarian cancer attributable to hyperglycemia, and more comprehensive epidemiological studies are needed in the future. In addition, the correlation between hyperglycemia and other confounding factors, such as obesity, needs to be figured out in the following research, and the effects of external intervention measures for hyperglycemia on the formation of ovarian cancer can be further discussed.

## Data availability statement

The original contributions presented in the study are included in the article/supplementary material, further inquiries can be directed to the corresponding authors.

## Author contributions

PW and QJ conceived the study, analyzed the data, and drafted the manuscript. XL constructed the idea and reviewed and edited the manuscript. LH planned the study, contributed to the discussion, and revised the manuscript. All authors read and approved the final manuscript.

## References

[1] Sung H Ferlay J Siegel RL Laversanne M Soerjomataram I Jemal A. Global cancer statistics 2020: GLOBOCAN estimates of incidence and mortality worldwide for 36 cancers in 185 countries. CA Cancer J Clin. (2021) 71:209–49. 10.3322/caac.21660 33538338

[2] Yunusova NV Kondakova IV Kolomiets LA. Afanas'ev SG, Kishkina AY, Spirina LV. The role of metabolic syndrome variant in the malignant tumors progression. Diabetes Metab Syndr. (2018) 12:807–12. 10.1016/j.dsx.2018.04.028 29699953

[3] Michels KA McNeel TS Trabert B. Metabolic syndrome and risk of ovarian and fallopian tube cancer in the United States: An analysis of linked SEER-Medicare data. Gynecol Oncol. (2019) 155:294–300. 10.1016/j.ygyno.2019.08.032 31495456 PMC6825892

[4] Tanha K Mottaghi A Nojomi M Moradi M Rajabzadeh R Lotfi S. Investigation on factors associated with ovarian cancer: an umbrella review of systematic review and meta-analyses. J Ovarian Res. (2021) 14:153. 10.1186/s13048-021-00911-z 34758846 PMC8582179

[5] Guo M Shang X Guo D. Metformin use and mortality in women with ovarian cancer: an updated meta-analysis. Int J Clin Pract. (2022) 2022:9592969. 10.1155/2022/9592969 35685604 PMC9159224

[6] Micha JP Rettenmaier MA Bohart RD Goldstein BH A. phase II, open-label, non-randomized, prospective study assessing paclitaxel, carboplatin and metformin in the treatment of advanced stage ovarian carcinoma. J Gynecol Oncol. (2022). 10.3802/jgo.2023.34.e15 36509462 PMC9995875

[7] Zhang D Zhao Y Wang T Xi Y Li N Huang H. Diabetes mellitus and long-term mortality of ovarian cancer patients. A systematic review and meta-analysis of 12 cohort studies. Diabetes Metab Res Rev. (2017) 33:4. 10.1002/dmrr.2868 27860198

[8] Wang L Zhong L Xu B Chen M Huang H. Diabetes mellitus and the risk of ovarian cancer: a systematic review and meta-analysis of cohort and case-control studies. BMJ Open. (2020) 10:e040137. 10.1136/bmjopen-2020-040137 33376163 PMC7778773

[9] Li G Zhang K Gong F Jin H A. study on changes and clinical significance of blood glucose, blood lipid and inflammation in patients with ovarian cancer. J BUON. (2019) 24:2322–6. 31983101

[10] Kellenberger LD Petrik J. Hyperglycemia promotes insulin-independent ovarian tumor growth. Gynecol Oncol. (2018) 149:361–70. 10.1016/j.ygyno.2018.02.003 29458977

[11] GBD 2019 Diseases and Injuries Collaborators. Global burden of 369 diseases and injuries in 204 countries and territories, 1990-2019: a systematic analysis for the Global Burden of Disease Study 2019. Lancet. (2020) 396:1204–22. 10.1016/S0140-6736(20)30925-9 33069326 PMC7567026

[12] Zhou Z Wang X Ren X Zhou L Wang N Kang H. Disease burden and attributable risk factors of ovarian cancer from 1990 to 2017: findings from the global burden of disease study 2017. Front Public Health. (2021) 9:619581. 10.3389/fpubh.2021.619581 34604147 PMC8484795

[13] Global Global Burden of Disease 2019 Cancer Collaboration Kocarnik JM Compton K Dean FE Fu W Gaw BL. Cancer incidence, mortality, years of life lost, years lived with disability, and disability-adjusted life years for 29 cancer groups from 2010 to 2019: a systematic analysis for the global burden of disease study 2019. JAMA Oncol. (2022) 8:420–44. 10.1001/jamaoncol.2021.6987 34967848 PMC8719276

[14] GBD Cancer Risk Factors Collaborators. The global burden of cancer attributable to risk factors, 2010-19: a systematic analysis for the Global Burden of Disease Study 2019. Lancet. (2022) 400:563–91. 10.1016/S0140-6736(22)01438-6 35988567 PMC9395583

[15] Devleesschauwer B Havelaar AH Maertens de Noordhout C Haagsma JA Praet N Dorny P. Calculating disability-adjusted life years to quantify burden of disease. Int J Public Health. (2014) 59:565–9. 10.1007/s00038-014-0552-z 24752429

[16] Parab J Sequeira M Lanjewar M Pinto C Naik G. Backpropagation neural network-based machine learning model for prediction of blood urea and glucose in CKD patients. IEEE J Transl Eng Health Med. (2021) 9:4900608. 10.1109/JTEHM.2021.3079714 34055499 PMC8159148

[17] Shao Y Wang Z Cao N Shi H Xie L Fu J. Prediction of 3-month treatment outcome of IgG4-DS based on BP artificial neural network. Oral Dis. (2021) 27:934–41. 10.1111/odi.13601 32790939

[18] GBD 2019 Respiratory Tract Cancers Collaborators. Global, regional, and national burden of respiratory tract cancers and associated risk factors from 1990 to 2019: a systematic analysis for the Global Burden of Disease Study 2019. Lancet Respir Med. (2021) 9:1030–49. 10.1016/S2213-2600(21)00164-8 34411511 PMC8410610

[19] Qiu H Cao S Xu R. Cancer incidence, mortality, and burden in China: a time-trend analysis and comparison with the United States and United Kingdom based on the global epidemiological data released in 2020. Cancer Commun (Lond). (2021) 41:1037–48. 10.1002/cac2.12197 34288593 PMC8504144

[20] Cabasag CJ Fagan PJ Ferlay J Vignat J Laversanne M Liu L. Ovarian cancer today and tomorrow: A global assessment by world region and Human Development Index using GLOBOCAN 2020. Int J Cancer. (2022) 151:1535–41. 10.1002/ijc.34002 35322413

[21] Lheureux S Gourley C Vergote I Oza AM. Epithelial ovarian cancer. Lancet. (2019) 393:1240–53. 10.1016/S0140-6736(18)32552-2 30910306

[22] Yoon H Lee S. Fatty acid metabolism in ovarian cancer: therapeutic implications. Int J Mol Sci. (2022) 23:4. 10.3390/ijms23042170 35216285 PMC8874779

[23] Baczewska M Bojczuk K Kolakowski A Dobroch J Guzik P Knapp P. Obesity and energy substrate transporters in ovarian cancer-review. Molecules. (2021) 26:6. 10.3390/molecules26061659 33809784 PMC8002293

[24] Zhang D Li N Xi Y Zhao Y Wang T. Diabetes mellitus and risk of ovarian cancer. A systematic review and meta-analysis of 15 cohort studies. Diabetes Res Clin Pract. (2017) 130:43–52. 10.1016/j.diabres.2017.04.005 28554142

[25] Vrachnis N Iavazzo C Iliodromiti Z Sifakis S Alexandrou A Siristatidis C. Diabetes mellitus and gynecologic cancer: molecular mechanisms, epidemiological, clinical and prognostic perspectives. Arch Gynecol Obstet. (2016) 293:239–46. 10.1007/s00404-015-3858-z 26338721

[26] Becker S Dossus L Kaaks R. Obesity related hyperinsulinaemia and hyperglycaemia and cancer development. Arch Physiol Biochem. (2009) 115:86–96. 10.1080/13813450902878054 19485704

[27] Adler AI Weiss NS Kamb ML Lyon JL. Is diabetes mellitus a risk factor for ovarian cancer? A case-control study in Utah and Washington (United States). Cancer Causes Control. (1996) 7:475–8. 10.1007/bf00052674 8813436

[28] Gallagher EJ LeRoith D. Diabetes, antihyperglycemic medications and cancer risk: smoke or fire? Curr Opin Endocrinol Diabetes Obes. (2013) 20:485–94. 10.1097/01.med.0000433065.16918.83 23974779

[29] Lheureux S Braunstein M Oza AM. Epithelial ovarian cancer: evolution of management in the era of precision medicine. CA Cancer J Clin. (2019) 69:280–304. 10.3322/caac.21559 31099893

[30] Chen Y Liu L Xia L Wu N Wang Y Li H. TRPM7 silencing modulates glucose metabolic reprogramming to inhibit the growth of ovarian cancer by enhancing AMPK activation to promote HIF-1alpha degradation. J Exp Clin Cancer Res. (2022) 41:44. 10.1186/s13046-022-02252-1 35101076 PMC8802454

[31] Gapstur SM Gann PH Lowe W Liu K Colangelo L Dyer A. Abnormal glucose metabolismand pancreatic cancer mortality. JAMA. (2000) 283:2552–8. 10.1001/jama.283.19.2552 10815119

[32] Hwang KA Park MA Kang NH Yi BR Hyun SH Jeung EB. Anticancer effect of genistein on BG-1 ovarian cancer growth induced by 17 b-estradiol or bisphenol A via the suppression of the crosstalk between estrogen receptor a and insulin-like growth factor-1 receptor signaling pathways. Toxicol Appl Pharmacol. (2013) 272:637–46. 10.1016/j.taap.2013.07.027 23933164

[33] Torre LA Trabert B DeSantis CE Miller KD Samimi G Runowicz CD. Ovarian cancer statistics, 2018. CA Cancer J Clin. (2018) 68:284–96. 10.3322/caac.21456 29809280 PMC6621554

[34] Webb PM Jordan SJ. Epidemiology of epithelial ovarian cancer. Best Pract Res Clin Obstet Gynaecol. (2017) 41:3–14. 10.1016/j.bpobgyn.2016.08.006 27743768

[35] Kim YE Jung YS Ock M Yoon SJ. DALY estimation approaches: understanding and using the incidence-based approach and the prevalence-based approach. J Prev Med Public Health. (2022) 55:10–8. 10.3961/jpmph.21.597 35135044 PMC8841194

[36] GBD 2019 Viewpoint Collaborators. Five insights from the global burden of disease study 2019. Lancet. (2020) 396:1135–59. 10.1016/S0140-6736(20)31404-5 33069324 PMC7116361

